# Combinatorial strategies for production improvement of anti-tuberculosis antibiotics ilamycins E_1_/E_2_ from deep sea-derived *Streptomyces atratus* SCSIO ZH16 Δ*ilaR*

**DOI:** 10.1186/s40643-022-00599-z

**Published:** 2022-10-22

**Authors:** Yunfei Zhu, Gaofan Zheng, Xiujuan Xin, Junying Ma, Jianhua Ju, Faliang An

**Affiliations:** 1 State Key Laboratory of Bioreactor Engineering, East China University of Science and Technology, Shanghai, 200237 China; 2 CAS Key Laboratory of Tropical Marine Bio-Resources and Ecology, Guangdong Key Laboratory of Marine Materia Medica, RNAM Center for Marine Microbiology, South China Sea Institute of Oceanology, Chinese Academy of Sciences, Guangzhou, China

**Keywords:** Ilamycins, *Streptomyces atratus*, Fermentation optimization, Exogenous adding strategy

## Abstract

Ilamycins E_1_/E_2_ are novel cyclic heptapeptides from *Streptomyces atratus* SCSIO ZH16, which have the MIC value of 9.8 nM against *Mycobacterium tuberculosis* H37Rv. However, the lower fermentative titer of ilamycins E_1_/E_2_ cut off further development for novel anti-TB lead drugs. In order to break the obstacle, the combinatorial strategy of medium optimization, fermentative parameters optimization, exogenous addition of metal ions, precursors, and surfactants was developed to promoted the production of ilamycins E_1_/E_2_. Addition of 1 mM ZnCl_2_ at 0 h, 1 g/L tyrosine at 96 h, and 2 g/L shikimic acid at 48 h increased the production of ilamycins E_1_/E_2_ from 13.51 to 762.50 ± 23.15, 721.39 ± 19.13, and 693.83 ± 16.86 mg/L, respectively. qRT-PCR results showed that the transcription levels of key genes in Embden–Meyerhof–Parnas pathway, hexose phosphate shunt pathway, and shikimic acid pathway were upregulated. In addition, the production of ilamycins E_1_/E_2_ reached 790.34 mg/L in a 5-L bioreactor by combinatorial strategy. Combinatorial strategies were used for improving ilamycins E_1_/E_2_ production in *S. atratus* Δ*ilaR* and provided a sufficient basis on further clinic development.

**Graphical Abstract:**

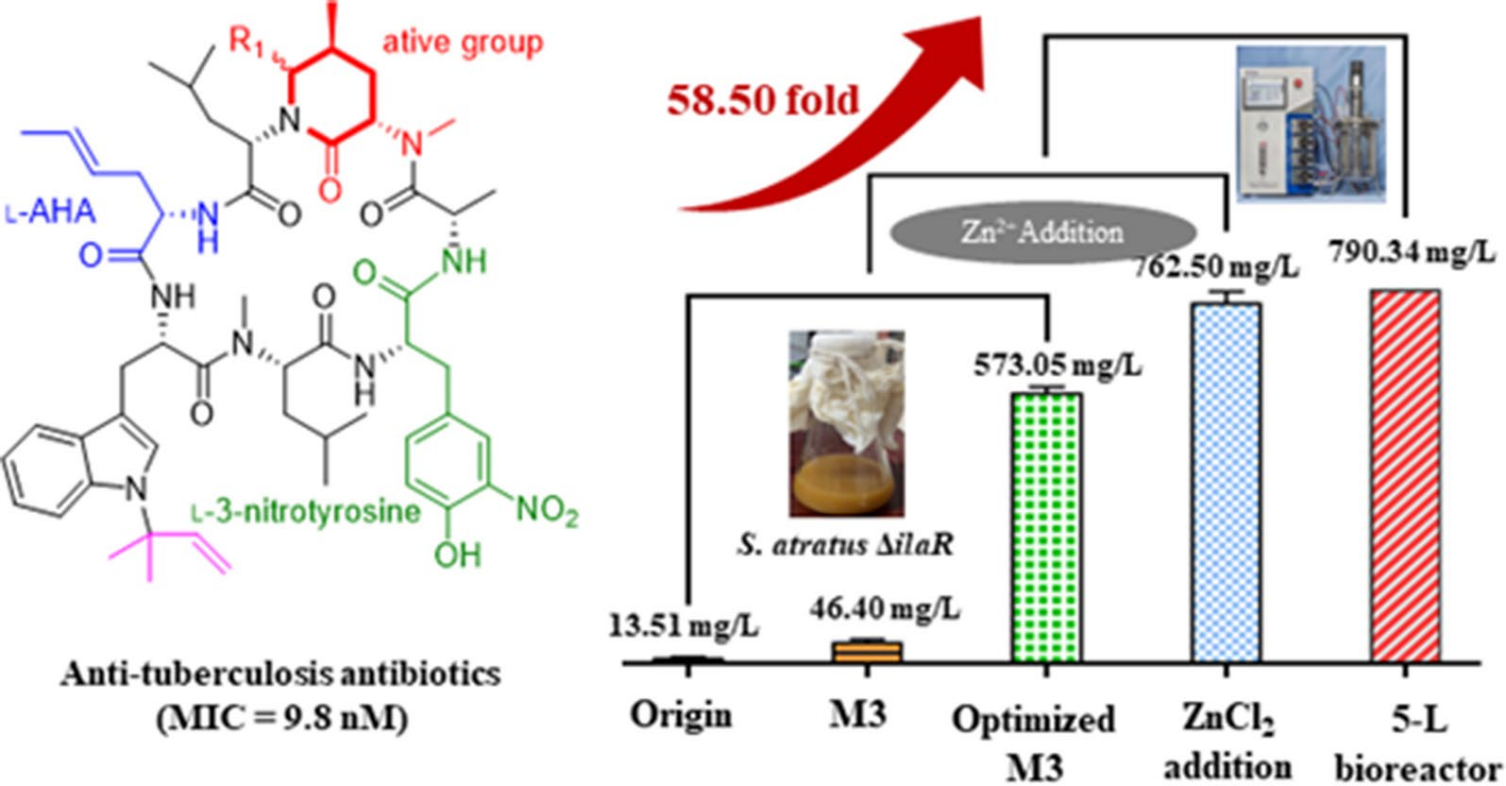

**Supplementary Information:**

The online version contains supplementary material available at 10.1186/s40643-022-00599-z.

## Introduction

Tuberculosis (TB), caused by pathogen *Mycobacterium tuberculosis*, ranks as the top infectious killer in the world, with the highest mortality even exceeding those from human immunodeficiency virus (HIV). Moreover, the prevention and control of TB have become more difficult because of the emergence and rapid dissemination of multidrug-resistant, extensively drug-resistant, and totally drug-resistant *M*. *tuberculosis* strains. In addition, after the development of rifampicin, few effective anti-TB agents were developed. The significant morbidity and mortality of TB, as well as its continuously emerging drug resistances, have posed great threats to the human health. Hence, novel anti-TB agents with increased potency and efficacy are urgently needed.

With the antibiotics abuse and the emergence of multidrug-resistance among human pathogenic bacteria, finding new antibiotics has become one of the important goals for drug research. Due to the unique marine physiological environment, marine actinomycetes, one of the main sources of marine microbial natural products, can produce much new secondary metabolites with potential bioactivity, which provide a new resource for drug research and development (Bull et al. 2000; Kamjam et al. 2017). In 2019, 145 new marine natural products were discovered from *Streptomyces*, accounting for 62.5% of bacterial sources, which indicated that *Streptomyces* played an important role in the mining of marine natural products (Carroll et al. 2021). In addition, it is estimated that more than 40% of the known microbial secondary metabolites are produced by actinomycetes, and more than 70% of the discovered natural antibiotics are from actinomycetes, such as gentamicin, erythromycin, cephalosporin and rifamycin (Dhakal et al. 2017; Emiliana et al. 2018). Up to now, more than 50% of antibiotics are developed from *Streptomyces*, which makes *Streptomyces* the main antibiotic producing microorganism in the pharmaceutical industry (Demain 2014).

**Fig. 1:**
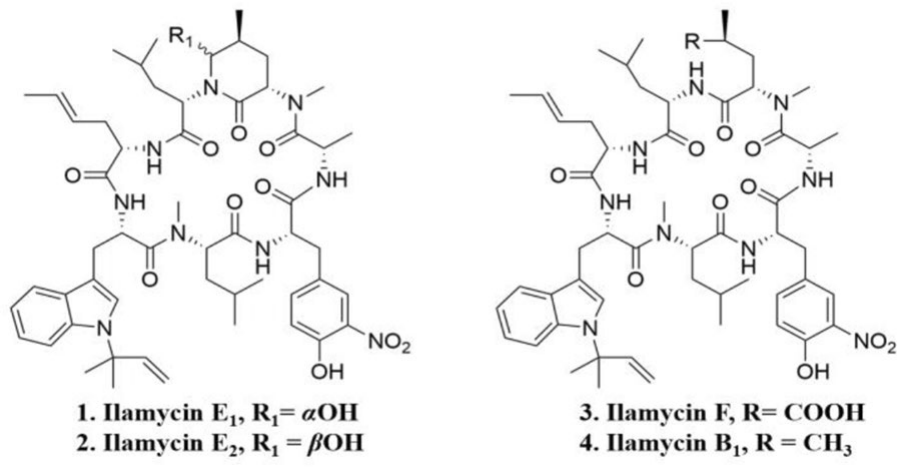
The structures of ilamycins

Ilamycins, also called rufomycins, are novel cyclic heptapeptides processing rare _*L*_-3-nitrotyrosine and _*L*_-2-amino-4-hexenoic acid (Cheng et al. 2018), which was first discovered by Takita T in *Streptomyces insulates* (Takita et al. 1962; Kazmaier and Junk 2021). Recently, six new ilamycins (B_1_, B_2_, C_1_, C_2_, D and E_1_) were isolated from a South China deep sea-derived strain *Streptomyces atratus* SCSIO ZH16 (Ma et al. 2017). In addition, ilamycins E_1_/E_2_/F/B_1_ (Fig. 1) are produced by the genetically engineered mutant strain (*S*. *atratus* SCSIO ZH16 Δ*ilaR*) (Ma et al. 2017), among which ilamycins E_1_/E_2_ have strong and selective anti-tuberculosis activity against *M*. *tuberculosis* H37Rv with the minimal inhibition concentration (MIC) value of 9.8 nM, which is 30-fold lower than that of the first-line anti-TB drug rifampicin (MIC 0.3 μM) (Zhou et al. 2019). However, the initial fermentation conditions of the strain producing ilamycins E_1_/E_2_ had only a production of 13.51 mg/L, which could not match the demands of new drug research and development. Although the total synthesis of ilamycins E1/E2 had been achieved afterwards, their synthetic route involved with more than 20 steps and less than 2% overall yield, apparently not satisfactory for industrial and clinical applications. Hence, it is an urgent requirement to scale-up the production of ilamycins E_1_/E_2_ though microbial fermentation.

Titer improvement is one of the main challenges in development of microbial natural products, especially in genetically engineered strains. Ilamycin-E1/E2 were accumulated by knocking out the IlaR (cytochrome P450) in biosynthesis gene cluster, which responsible for the epoxidation of isopentene moiety in ilamycins. The inactivation of *ilaA* and overexpression of *ilaB* increased the total production of ilamycins to 3.0- and 1.9-fold compared to those in the wild-type *S*. *atratus* SCSIO ZH16, respectively (He et al. 2020). Medium optimization and subsequent fermentative regulation strategy enabled the scaled-up production of ilamycins E_1_/E_2_ to 415.7 ± 29.2 mg/L in *S*. *atratus* SCSIO ZH16 Δ*ilaR* in a 300-L bioreactor (Fan et al. 2022). However, there are few reports of titer enhancement of ilamycins E_1_/E_2_ by fermentative process parameters optimization in wild-type strains and their mutants, especially.

For a newly isolated wild strain, it is essential to enhance the titer of secondary metabolites by optimizing the fermentation process parameters, which includes medium, fermentation conditions, exogenous addition of metal ions, precursors, surfactants and other substances (Tao et al. 2019). For instance, Duan et al. optimized the culture and fermentation conditions for *Streptomyces* ma. *FS-4* by single-factor and response surface experiments, and the diameter of bacteriostasis circle for *Bacillus subtilis* increased from 16.5 to 26.7 mM (Duan et al 2020). Jia et al. added 2 mM Mg^2+^ to the culture medium to make the maximum production of ansamitocin P-3 with 85 mg/L, which is 3 times higher than that of the control group (Jia and Zhong, 2011). Khushboo et al. added precursor _*L*_- leucine to enhance the production of lipstatin, a secondary metabolite of *Streptomyces toxytricini*, from 5.011 to 5.765 mg/g (Khushboo et al. 2020). Zhang et al. made the production of monacolin K reached 2026.0 ± 30.4 mg/L through adding 40 g/L Triton X-100, which was 84.9% higher than that of the control (Zhang et al. 2014).

In this study, we firstly provided a combinatorial strategy of medium optimization, fermentative parameters optimization, exogenous addition of metal ions, precursors, and surfactants to enhance the production of ilamycins E_1_/E_2_. After optimizing the fermentation medium and parameters, exogenous adding ZnCl_2_, tyrosine, shikimic acid, and sorbitol to medium, the fermentative titer of ilamycins E_1_/E_2_ was proved from 13.51 to 762.50 ± 23.15 mg/L with 56.4-fold. The RT-PCR results showed that transcription level of eight genes (*G6PD*, *6-PFK*, *PK*, *6-PDGH*, *DAHPS*, *CS*, *SKD*, *3-DHQS*) related to biosynthesis of ilamycins was promoted by addition of ZnCl_2_, tyrosine, shikimic acid and sorbitol, respectively. Exogenous addition could effectively regulate the balance between primary and secondary metabolism and enhance the production of ilamycins. Last but not least, the fermentation was optimized and scaled up to a 5-L bioreactor, in which the highest ilamycins E_1_/E_2_ production reached 790.34 mg/L. To the best of our knowledge, this is the highest titer reported in published literatures. This work will provide a valuable basis for industrial pilot production of anti-tuberculosis lead drug ilamycins E1/E2.

## Experimental

### Microorganism, media and fermentative conditions

*S*. *atratus* SCSIO ZH16 Δ*ilaR* is a genetically engineered mutant strain of *S*. *atratus* SCSIO ZH16 (CGMCC No.12198), which was provided by Ma's laboratory (South China Sea Institute of Oceanology, Chinese Academy of Sciences, Guangzhou, China) and its spores were stored in 20% (v/v) glycerol at − 80 °C for long-term storage. *S*. *atratus* SCSIO ZH16 Δ*ilaR* was cultured on an agar plate (yeast extract 4 g/L, malt extract 10 g/L, soluble starch 4 g/L and oat 10 g/L) with 0.03 mM apramycin at 28 °C for 5–7 days. A piece of agar (about 1 cm^2^) was dug and transferred into a 250-mL Erlenmeyer flask with 25 mL seed medium (glucose 20 g/L, peptone 2 g/L, yeast extract 2 g/L, soybean meal 5 g/L, MgSO_4_·7H_2_O 0.5 g/L, KH_2_PO_4_ 0.5 g/L, NaCl 4 g/L, CaCO_3_ 2 g/L, sea salt 30 g/L) for culture at 200 rpm, 28 °C for 60 h.

### Carbon and nitrogen sources optimization of initial medium

Seven kinds of media for actinomycetes fermentation that could be used for target compounds production are shown in Additional file 1: Table S1. 10% (v/v) seed medium was inoculated into a 250-mL Erlenmeyer flask containing 25 mL fermentation medium at 200 rpm, 30 °C for 168 h. To screen out the suitable carbon and nitrogen source for ilamycins E_1_/E_2_ production, five carbon sources (glucose, maltose, sucrose, mannitol and soluble starch) and six nitrogen sources (soybean meal, yeast extract paste, yeast extract powder, peptone, malt extract and beef extract) were screened, respectively.

### Plackett–Burman design (PDB) based medium optimization

PBD was carried out to screen the optimal fermentation medium (soluble starch 120 g/L, soybean meal 20 g/L, corn steep liquor 2.4 g/L, NaCl 6 g/L, NaNO_3_ 9.6 g/L, KH_2_PO_4_ 0.24 g/L, (NH_4_)_2_SO_4_ 6.4 g/L, CaCO_3_ 9.6 g/L), and three main factors (*p* < 0.05) were selected, which affected mostly the production of ilamycins E_1_/E_2_. 8 variables and 12 groups of experiments (Additional file 1: Tables S2 and S3) were carried out by using Design Expert 12 software (Stat-Ease, MN, USA), and each variate had two levels, with codes of  −  1 and + 1. After obtaining three main factors, a single-factor gradient experiment was conducted to determine their more suitable concentration, while the concentration of other components remained unchanged. Based on the single-factor gradient experiment, the central composite design (CCD) was carried out by using Design Expert 12 software. Five concentration levels (−  1.68, −  1, 0, 1, 1.68) were set for three main factors, and 20 groups of experiments were carried out according to different levels of the main factors, which are listed in Additional file 1: Tables S4 and S5. The results of CCD conformed to the following second-order polynomial equation:

$$Y={\beta }_{0}+{\sum }_{i=1}^{3}{\beta }_{i}{X}_{i}+{\sum }_{i=1}^{3}{\beta }_{ii}{X}_{i}^{2}+\sum_{1\le i<j}^{3}{\beta }_{ij}{X}_{i}{X}_{j},$$

where *Y* represents the predicted response; ${\beta }_{0}$, ${\beta }_{i}$, ${\beta }_{ii}$ and ${\beta }_{ij}$ represent the constant coefficients; and ${X}_{i}$ and ${X}_{j}$ represent the independent variables.

### Optimization of fermentation parameters

The initial pH value of fermentation medium (4, 5, 6, 7, 8, 9, 10), culture temperature (24 °C, 26 °C, 28 °C, 30 °C, 32 °C, 34 °C), inoculation amount (1%, 5%, 10%, 15%, 20%), inoculation time (24 h, 36 h, 48 h, 60 h, 72 h, 84 h, 96 h), flask shape (Erlenmeyer flask, Erlenmeyer baffled flask), volume (25 mL, 50 mL, 75 mL, 100 mL), rotational speed (150 rpm, 175 rpm, 200 rpm, 225 rpm, 250 rpm), and culture time (24 h, 48 h, 72 h, 96 h, 120 h, 144 h, 168 h, 192 h, 216 h, 240 h) were tested to determine the best condition while other parameters remained unchanged.

### Evaluation and optimization of exogenous additions

The effects of seven metal ions (Al^3+^, Fe^3+^, Mg^2+^, Mn^2+^, Zn^2+^, Ca^2+^, Cu^2+^) on the production of ilamycins E_1_/E_2_ were investigated. Initial adding concentration of seven chlorinated salts, which were filtered by 0.22 μm membrane and added at 72 h, was set at 1 mM. After screening the optimal chloride salt, the concentration and adding time were evaluated. Considering the balance between primary and secondary metabolism, the effects of seven amino acids (tyrosine, phenylalanine, tryptophan, 3-nitro-tyrosine, leucine, N-methyl-leucine and alanine) and shikimic acid on the production of ilamycins E_1_/E_2_ were investigated. The initial adding concentration of seven amino acids and shikimic acid, which were filtered by 0.22 μm membranes and added after fermentation for 72 h, was set at 0.5 g/L. After selecting out the suitable substance that could increase the production of ilamycins E_1_/E_2_, the concentration and time of addition were screened. To elevated oxygen transfer efficiency, the effects of six surfactants (Tween-80, Triton X-100, sorbitol, sodium dodecyl sulfate, hexadecyl trimethyl ammonium bromide and dimethyl sulfoxide) on the production of ilamycins E_1_/E_2_ were investigated. The addition concentration of 6 surfactants was set at 1 g/L, filtered by 0.22 μm membranes and added after fermentation for 144 h. After determining the optimal surfactant, the concentration and time of addition were then screened. The viscosity of fermentation liquor was measured by Brookfield DV-II + PRO Rotational Viscometer (Brookfield Co., MA, USA).

### Transcription-level analysis of key genes by qRT-PCR

Based on the biosynthetic pathway analysis of ilamycins E_1_/E_2_, eight genes (*G6PD*, *6-PFK*, *PK*, *6-PDGH*, *DAHPS*, *CS*, *SKD*, *3-DHQS*) corresponding to key enzymes of the metabolism pathways and the reference gene (*SCO1544*) were selected (Li et al. 2015). The total RNA of *S*. *atratus* SCSIO ZH16 Δ*ilaR* was extracted by MolPure® Bacterial RNA Kit (Yeasen Biotechnology Co., Ltd., Shanghai, China), and then reverse-transcribed by Hifair® III 1st Strand cDNA Synthesis SuperMix for qPCR (Yeasen Biotechnology (Shanghai) Co., Ltd., Shanghai, China). Subsequently, according to Hieff® qPCR SYBR Green Master Mix, the transcriptional levels of *G6PD*, *6-PFK*, *PK*, *6-PDGH*, *DAHPS*, *CS*, *SKD*, *3-DHQS*, and *SCO1544* were determined. The process of qRT-PCR was briefly described as follows: the pre-denature stage (95 °C for 5 min), the denature stage (95 °C for 10 s), the annealing stage (60 °C for 20 s), and the extension stage (72 °C for 30 s). There are 40 cycles of reactions in the three stages of denature, annealing and extension. The dissolution curve phase uses the default settings of the instrument. The sequences of forward/reverse primers of target genes are listed in Additional file 1: Table S6.

### Fermentative production of ilamycins E1/E2 in 5-L bioreactor

After medium optimization in flask level, the fermentative production of ilamycins E1/E2 was validate in 5-L bioreactor (Shanghai Guoqiang Biochemical Engineering Equipment Co., Ltd., Shanghai, China) by optimization of initial pH value and DO level. In addition, ZnCl_2_ addition strategy was also carried out in this bioreactor. The aeration rate and tank pressure were kept at 1.0 vvm and 0.03 Mpa, respectively. 0.3% defoamer was added, and the linkage of rotational speed and dissolved oxygen was set. The rotational speed ranged from 200 to 600 rpm. When the dissolved oxygen level was lower than 20%, the rotational speed was increased by 10 rpm to keep the dissolved oxygen between 0 and 60%. The level of residual sugar in fermentation broth was determined by 3,5-dinitrosalicylic acid colorimetry (DNS method) (Wu et al. 2018).

### Ilamycins extraction and quantitative analysis by HPLC

The fermentation broth and mycelium were separated by vacuum filtration. The supernatant was extracted with ethyl acetate (EtOAc) for three times and then evaporated under vacuum to dryness at 45 °C. After dissolving with methanol (MeOH), the solution was transferred to a centrifugal tube, centrifuged at 9000 rpm. It was filtered by 0.22-μm membranes and stored at 4 °C for HPLC detection. The filtered mycelium was ultrasonically extracted with MeOH and the solution was then concentrated under vacuum to obtain extract at 45 °C. After dissolving with MeOH, the solution was transferred to a centrifugal tube, centrifuged at 9000 rpm. It was filtered by 0.22 μm membranes and stored at 4 °C for HPLC detection. The ilamycins E_1_/E_2_/F/B_1_ standards were dissolved in MeOH and determined by HPLC instrument (Agilent Technologies 1260 Infinity, CA, USA) on the Agilent ZORBAX Eclipse XDB-C18 column (4.6 mm × 150 mm, 5 μm) to obtain standard curves. HPLC conditions are as follows: solvent system (solvent A, 0.1% acetic acid aqueous solution; solvent B, acetonitrile); 15% B to 71% B (0–10 min, linear gradient), 71% B to 90% B (10–11.5 min, linear gradient), 90% B (11.5–25 min), 90% B to 15% B (25–27.1 min, linear gradient), 15% B (27.1–30 min); the flow rate was set as 1 mL/min; the ultraviolet detection was at 280 nm. As ilamycins E_1_/E_2_ are epimers and can be transformed into each other, the standard curve measured by ilamycins E_1_ was used for the quantification of the total ilamycins E_1_/E_2_. The standard curve of ilamycins E_1_ adopted linear equation: $Y=9.088X-96.83$ (*R*^*2*^ = 0.9987). The standard curve of ilamycin F adopted linear equation: $Y=6.153X-19.28$ (*R*^*2*^ = 0.9988). The standard curve of ilamycin B_1_ adopted linear equation: $Y=11.89X-102.07$ (*R*^*2*^ = 0.9998). $X$ is the peak area of each ilamycin, and $Y$ is the concentration of each ilamycin. All experiments were carried out in triplicate.

### Statistical analysis

All experimental results were presented as the mean ± standard deviation for three replicates. Data analysis was performed using a one-way analysis of variance (ANOVA) with the least significant difference test using the standard of *p* < 0.05 by Origin 9.0 software.

## Results and discussion

### High-production medium exploration of ilamycins E_1_/E_2_ by PD and CCD

**Fig. 2:**
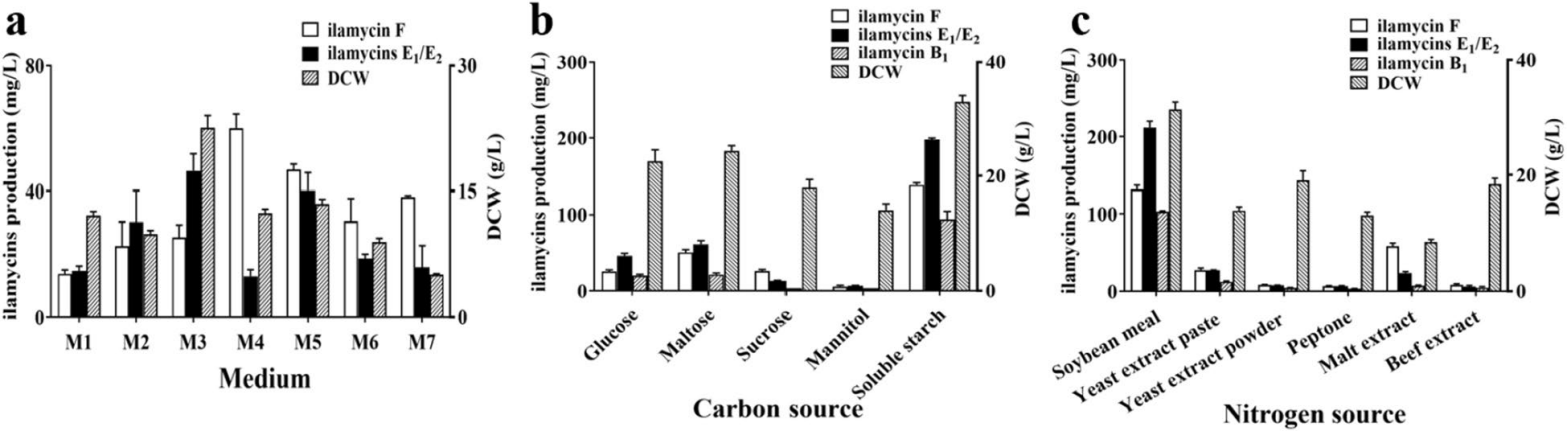
Ilamycins production and DCW in M1-M7 (**a**) and effect of different carbon (**b**) and nitrogen (**c**) sources on DCW and ilamycins production

Suitable culture and fermentative media are crucial to biosynthesis of secondary metabolites for wild and engineered strains. Thus, 7 commonly used fermentation media for actinomycetes and streptomyces were selected and tested for ilamycins production as shown in Additional file 1: Table S1. As shown in Fig. 2a, among those media, the highest ilamycins E_1_/E_2_ production (46.40 ± 5.41 mg/L) was obtained from M3 medium. Thus, M3 medium was selected as the initial fermentation medium of *S*. *atratus* SCSIO ZH16 Δ*ilaR*. Carbon and nitrogen sources were basic and essential in medium to biosynthesis secondary metabolites. Next, the traditional single-factor experiment was used to further optimize carbon and nitrogen sources. Five kinds of carbon sources (glucose, maltose, sucrose, mannitol and soluble starch) and six kinds of nitrogen sources (soybean meal, yeast extracted paste, yeast extract powder, peptone, malt extract, and beef extract) were screened under the change principle of same concentration with those of M3. The results showed that soluble starch and soybean meal were the better combination, and the production of ilamycins E_1_/E_2_ reached 211.91 ± 14.18 mg/L (Fig. 2b, c).

**Fig. 3:**
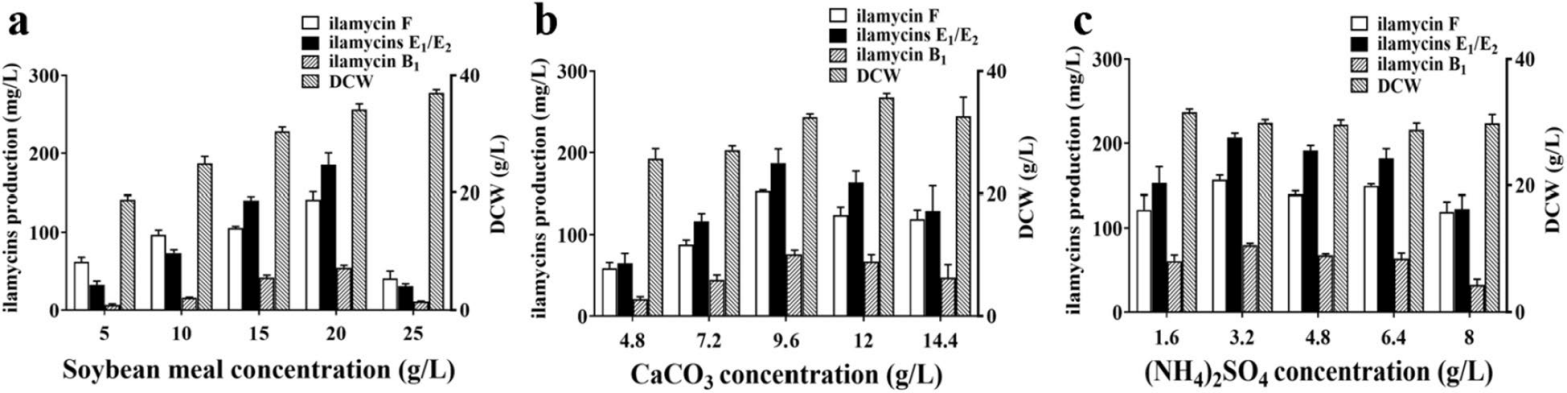
Effect of soybean meal (**a**), CaCO_3_ (**b**), and (NH_4_)_2_SO_4_ (**c**) concentrations on DCW and ilamycins production

Compared with the traditional single-factor experiment, the PB factorial design can effectively reduce experimental trials and quickly confirm the contribution of each component. The PBD and CCD were adopted by using Design Expert 12 software to further effectively optimize the fermentation medium. First of all, the experiment of PBD with 12 experimental trials, were used to assess influence of the eight components including soluble starch, soybean meal, and corn steep liquor, NaNO_3_, (NH4)_2_SO_4_, NaCl, KH_2_PO_4_, CaCO_3_, with two concentration levels in medium on the production of ilamycins E1/E2 (Additional file 1: Tables S2 and S3). The results shown in Table 1 remind that the mathematical model matched well, and three factors (*p* < 0.05) that had remarkable effects on the production of ilamycins E_1_/E_2_ in the fermentation medium were screened out: soybean meal (X_2_), CaCO_3_ (X_8_) and (NH_4_)_2_SO_4_ (X_5_). The following selection on gradient experiment of each single factor showed the center point: soybean meal 20 g/L, CaCO_3_ 9.6 g/L, and (NH_4_)_2_SO_4_ 3.2 g/L (Fig. 3).

**Table 1:** Results of PBD ANOVA analysis

Source	Sum of squares	df	Mean square	*F*-value	*p*-value	Contribution	
Model	24989.57	8	3123.70	15.30	0.0233		Significant
X_1_	1383.98	1	1383.98	6.78	0.0801	5.41	
X_2_	10922.97	1	10922.97	53.51	0.0053	42.66	
X_3_	947.59	1	947.59	4.64	0.1202	3.70	
X_4_	78.63	1	78.63	0.39	0.5788	0.31	
X_5_	4763.83	1	4763.83	23.34	0.0169	18.61	
X_6_	13.39	1	13.39	0.066	0.8144	0.052	
X_7_	46.00	1	46.00	0.23	0.6674	0.18	
X_8_	6833.19	1	6833.19	33.47	0.0103	26.69	
Residual	612.40	3	204.13				
Cor total	25601.97	11

After the center point was determined, the effect of three significant factors with five concentration levels and 20 groups of experimental results on ilamycins E_1_/E_2_ production are shown in Additional file 1: Tables S4 and S5. The regression model was significant, and the lack of fit was not consequential as Table 2 shows. The determination coefficient *R*^*2*^ (*R*^*2*^ = 0.9566) and adjustment determination coefficient Adj*R*^*2*^ (Adj*R*^*2*^ = 0.9176) were both greater than 90%, and the relative deviation *CV* was low (*CV* = 7.67%), which meant that the model could well analyze and predict the response value. The experimental results were fitted by multiple quadratic regression, and the equation was obtained as: $Y=198.05+13.06A+39.37B+20.19C+4.15AB$ $+1.05AC+6.85BC-12.05{A}^{2}-12.70{B}^{2}-17.69{C}^{2}$.The highest production of ilamycins E_1_/E_2_ predicted by Design Expert 12 software was 251.70 mg/L with the addition of three significant factors: soybean meal (23.49 g/L), CaCO_3_ (17.67 g/L), and (NH_4_)_2_SO_4_ (4.68 g/L). Follow-up experiments were carried out according to the above formula three times, with three groups in parallel each time. The experimental production was 255.52 mg/L, which was a good match to the predicted value. The result proved the reliability of the regression equation and the effectiveness of the statistical method.

**Table 2:** Results of CCD ANOVA analysis

Source	Sum of Squares	df	Mean Square	*F*-value	*p*-value	
Model	37,105.50	9	4122.83	24.50	< 0.0001	Significant
A	2327.73	1	2327.73	13.83	0.0040	
B	21,165.21	1	21,165.21	125.79	< 0.0001	
C	5567.39	1	5567.39	33.09	0.0002	
AB	138.09	1	138.09	0.8207	0.3863	
AC	8.78	1	8.78	0.0522	0.8240	
BC	375.67	1	375.67	2.23	0.1660	
A^2^	2091.52	1	2091.52	12.43	0.0055	
B^2^	2323.76	1	2323.76	13.81	0.0040	
C^2^	4510.39	1	4510.39	26.81	0.0004	
Residual	1682.54	10	168.25			
Lack of fit	1203.06	5	240.61	2.51	0.1678	Not significant
Pure error	479.48	5	95.90			
Cor total	38,788.04	19				

### Optimization of fermentation parameters

**Fig. 4:**
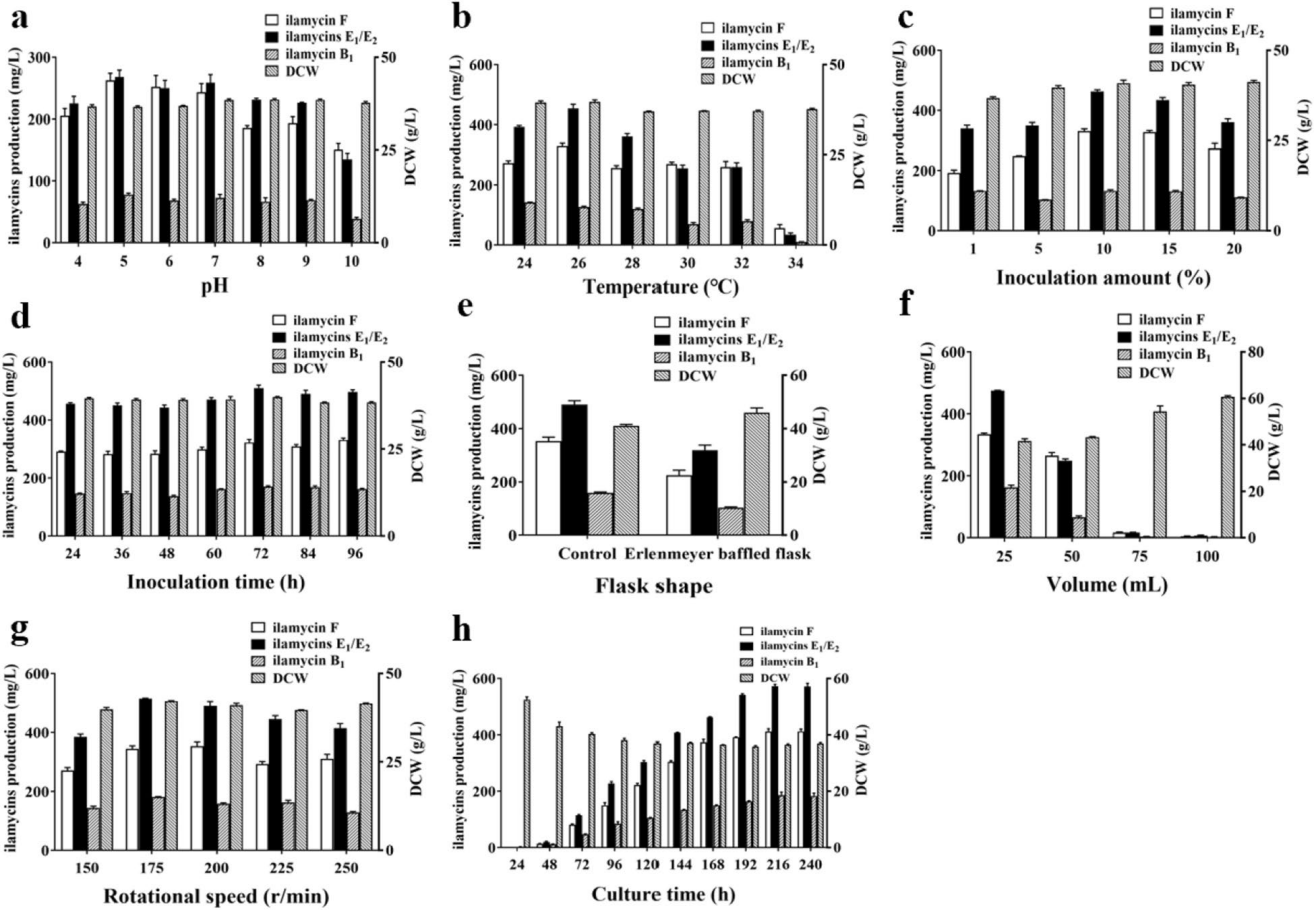
Effect of initial pH value (**a**), temperature (**b**), inoculation amount (**c**), inoculation time (**d**), flask shape (**e**), volume (**f**), rotational speed (**g**), and culture time (**h**) on DCW and ilamycins production

As is known, fermentation parameters, such as pH value, temperature, inoculation amount, fermentation volume, rotational speed, often affect secondary metabolism of microorganisms. Here, the initial pH value of each experimental group was controlled from 4.0 to 10.0 by NaOH solution. As shown in Fig. 4a, the culture medium with the initial pH value of 5.0 achieved the high production of ilamycins E_1_/E_2_. Temperature is an important factor for actinomycetic growth and metabolism and that of each experimental group was controlled from 24 °C to 34 °C. As shown in Fig. 4b, the suitable temperature of culture medium is 26 °C. Inoculation amount and inoculation time is the circumstantial evidence of seed quality, which is also a factor affecting the fermentation. Besides, dissolved oxygen always affects actinomycetic growth and metabolism, thus, inoculation amount (Fig. 4c), inoculation time (Fig. 4d), flask shape (Fig. 4e), liquid volume (Fig. 4f), rotational speeds (Fig. 4g) and culture time were tested to qualitatively evaluate the influence of seed and oxygen on the production of ilamycins E_1_/E_2._ The production of ilamycins E_1_/E_2_ reached the high production 573.05 ± 11.33 mg/L under the following better fermentation conditions as: the fermentation medium contained soluble starch 120 g/L, soybean meal 23.49 g/L, corn steep liquor 2.4 g/L, NaCl 6 g/L, NaNO_3_ 9.6 g/L, KH_2_PO_4_ 0.24 g/L, (NH_4_)_2_SO_4_ 4.68 g/L, CaCO_3_ 17.67 g/L; the initial pH value was 5; the inoculation amount was 10% after incubation for 72 h; the volume was 25 mL in a 250-mL Erlenmeyer flask; the rotational speed was 175 rpm; the fermentation temperature was 26 °C; the culture time was 216 h. These results implied that a mild oxygen level, suitable pH, temperature values and high seed quality could promote the production of ilamycins E_1_/E_2_.

### Enhanced production of ilamycins E_1_/E_2_ by co-addition strategy

#### Metal ions addition

**Fig. 5:**
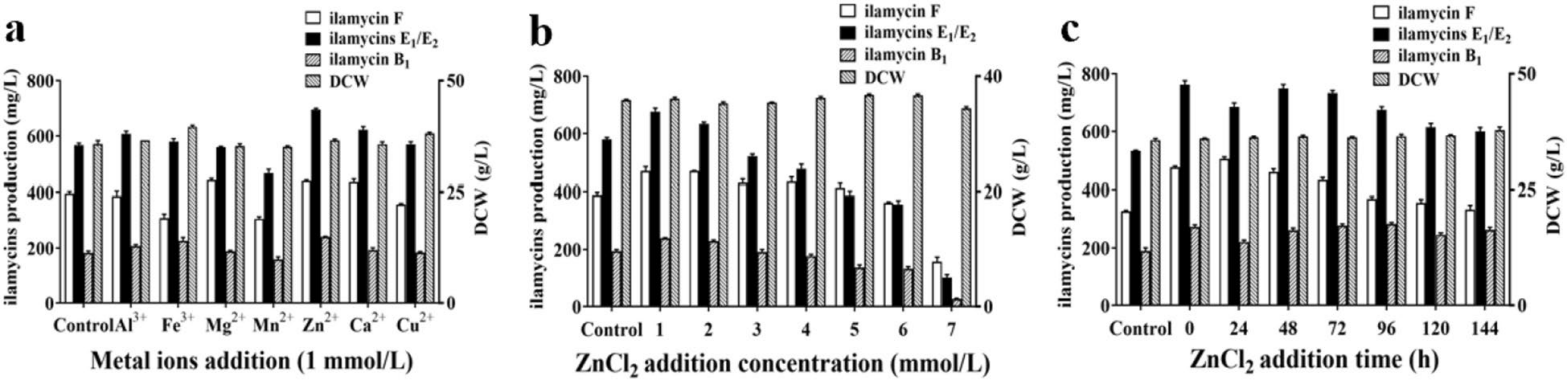
Effect of metal ions addition (**a**), ZnCl_2_ addition concentration (**b**), and ZnCl_2_ addition time (**c**) on DCW and ilamycins production

Addition of metal ions as an efficient fermentation strategy are used to regulate secondary metabolites biosynthesis and cellular metabolism, which involves in ionic balance, enzyme activity, signal transduction and metabolites biosynthesis. Among the seven selected chlorinated salts, ZnCl_2_ showed obvious effects on the production of ilamycins E_1_/E_2_ as shown in Fig. 5a. When adding 1 mM ZnCl_2_ solution at 0 h, the production of ilamycins E_1_/E_2_ reached 762.50 ± 23.15 mg/L as shown in Fig. 5b, c. Although the content of metal ions is very low in microbial cells, they play important effects on the structure formation, cell growth and metabolism.

#### Precursors addition

**Fig. 6:**
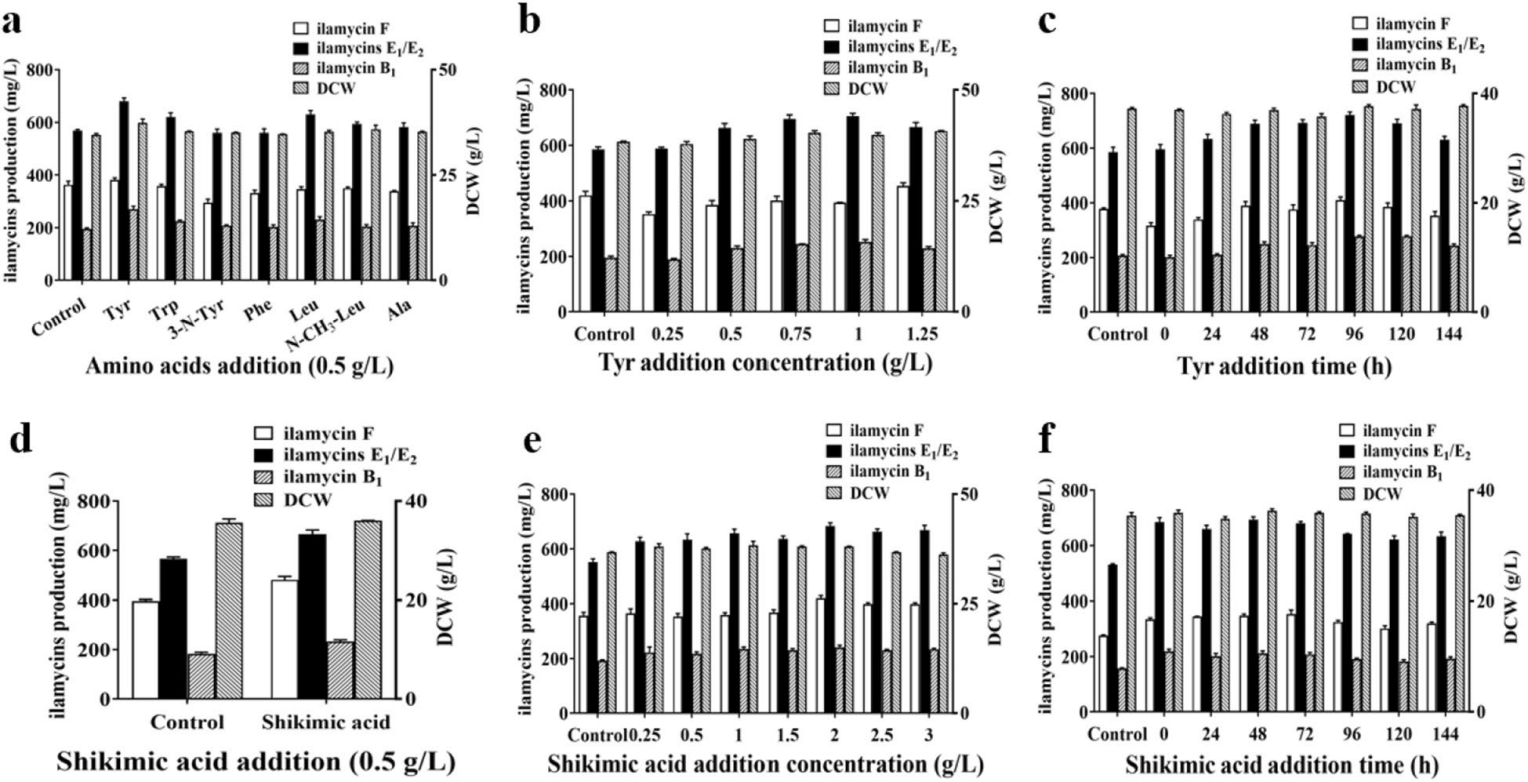
Effect of amino acids addition (**a**), Tyr addition concentration (**b**), Tyr addition time (**c**), shikimic acid addition (**d**), shikimic acid addition concentration (**e**), and shikimic acid addition time (**f**) on DCW and ilamycins production

Precursor addition is the frequent and effective method for enhancement of secondary metabolites in laboratory. Based on the biosynthetic pathway analysis of ilamycins, seven kinds of amino acids (tyrosine, phenylalanine, tryptophan, 3-nitro-tyrosine, leucine, N-methyl-leucine and alanine) and shikimic acid were selected and tested. The results showed that the addition of tyrosine and shikimic acid could promote the production of ilamycins (Fig. 6a, d). Adding 1 g/L Tyr at 96 h and 2 g/L shikimic acid at 48 h could make the production of ilamycins E_1_/E_2_ reach to 721.39 ± 19.13 mg/L and 693.83 ± 16.86 mg/L, respectively (Fig. 6b–f).

### Measurement of transcription level of the key enzyme genes by qRT-PCR

**Fig. 7:**
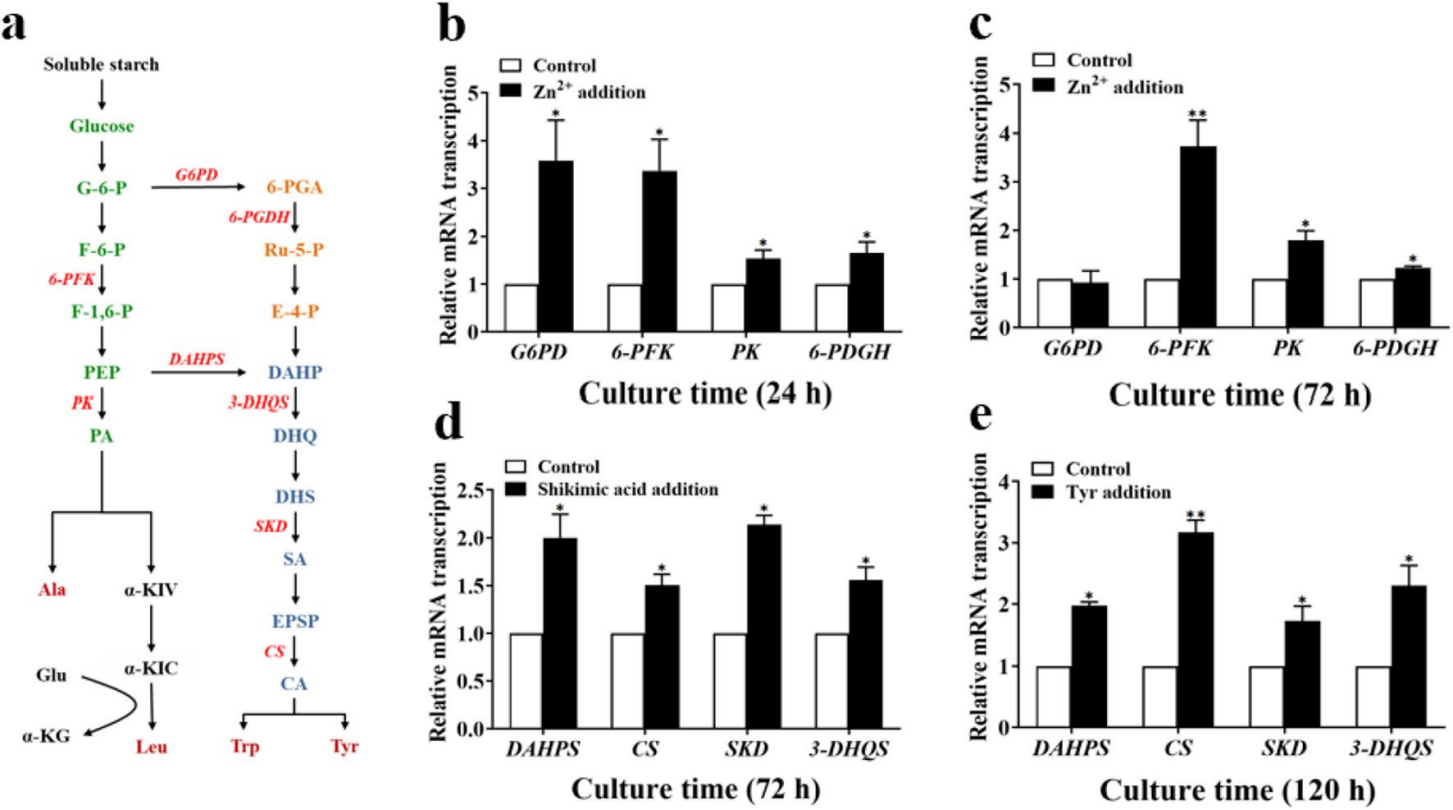
The metabolic pathway of ilamycins synthesis precursors (**a**) and comparison between the transcriptional levels of target genes after ZnCl_2_ (**b**, **c**), shikimic acid (**d**), Tyr (**e**) addition

Four kinds of amino acids (tyrosine, tryptophan, leucine and alanine) were reported involved in the ilamycins biosynthesis. The biosynthetic pathways of these four amino acids were Embden–Meyerhof–Parnas (EMP) pathway, hexose phosphate shunt (HMS) pathway, and shikimic acid pathway as Fig. 7a shows. The transcription levels of eight genes (*G6PD*, *6-PFK*, *PK*, *6-PDGH*, *DAHPS*, *CS*, *SKD*, *3-DHQS*) corresponding to key enzymes in the pathways were detected after adding ZnCl_2_, Tyr, and shikimic acid. The results in Fig. 7b show that the transcription levels of *G6PD*, *6-PFK*, *PK*, *6-PDGH* genes were increased by 3.58-, 3.37-, 1.54- and 1.65-fold, respectively, in the ZnCl_2_ addition group after 24 h fermentation. Furthermore, the transcription levels of *6-PFK*, *PK* and *6-PDGH* genes were increased by 3.72-, 1.80- and 1.23-fold, respectively, after 72 h fermentation (Fig. 7c), which was more conducive to the amino acid precursors synthesis. When shikimic acid was added in broth at 72 h, the transcription levels of *DAHPS*, *CS*, *SKD* and *3-DHQS* genes were increased by 2.00-, 1.50-, 2.14- and 1.56-fold, respectively (Fig. 7d). And the transcription levels of *DAHPS*, *CS*, *SKD* and *3-DHQS* genes were also increased by 1.98-, 3.17- 1.73- and 2.30-fold, respectively, with the addition of Tyr after fermentation 120 h (Fig. 7e).

#### Surfactants addition

**Fig. 8:**
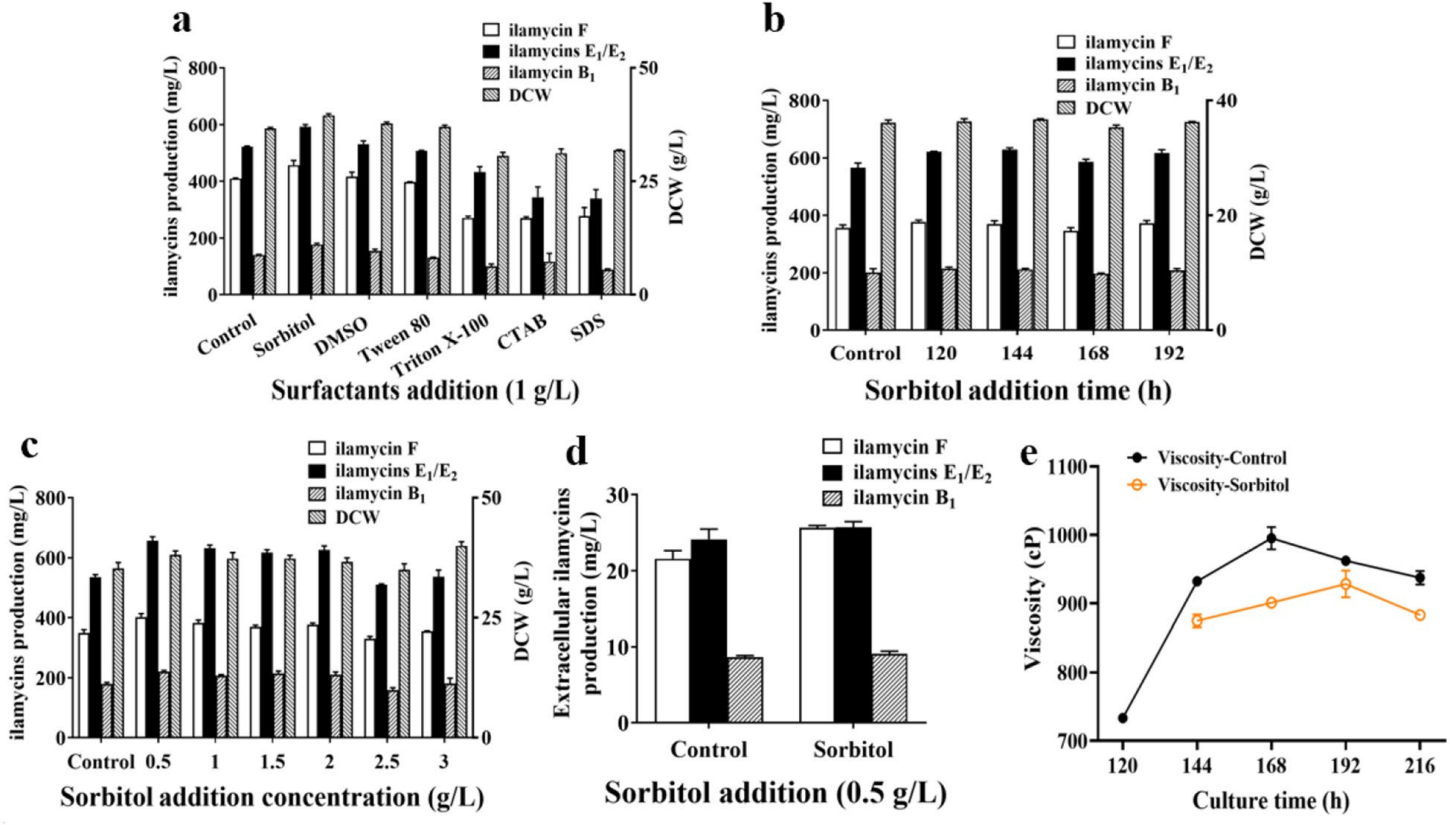
Effect of surfactants addition (**a**), sorbitol addition time (**b**), sorbitol addition concentration (**c**) on DCW and ilamycins production, and effect of sorbitol addition on extracellular ilamycins production (**d**) and viscosity (**e**)

Among the detected six kinds of surfactant, including nonionic-type (Tween-80, Triton X-100, sorbitol), ionic-type (sodium dodecyl sulfate, hexadecyl trimethyl ammonium bromide), and special surfactant (dimethyl sulfoxide), 0.5 g/L sorbitol addition at 144 h could enhance the production of ilamycins E_1_/E_2_ to 656.96 ± 22.68 mg/L (Fig. 8a–c). Figure 8d, e shows that the addition of sorbitol could not promote the efflux of ilamycins, but reduced the viscosity of the fermentation broth and improved the transmission and diffusion of oxygen. Therefore, the appropriate surfactants can improve the transmission and diffusion of oxygen in the fermentation broth, which is essential for fermentation in bioreactor.

### Fermentation in 5-L bioreactor

**Fig. 9:**
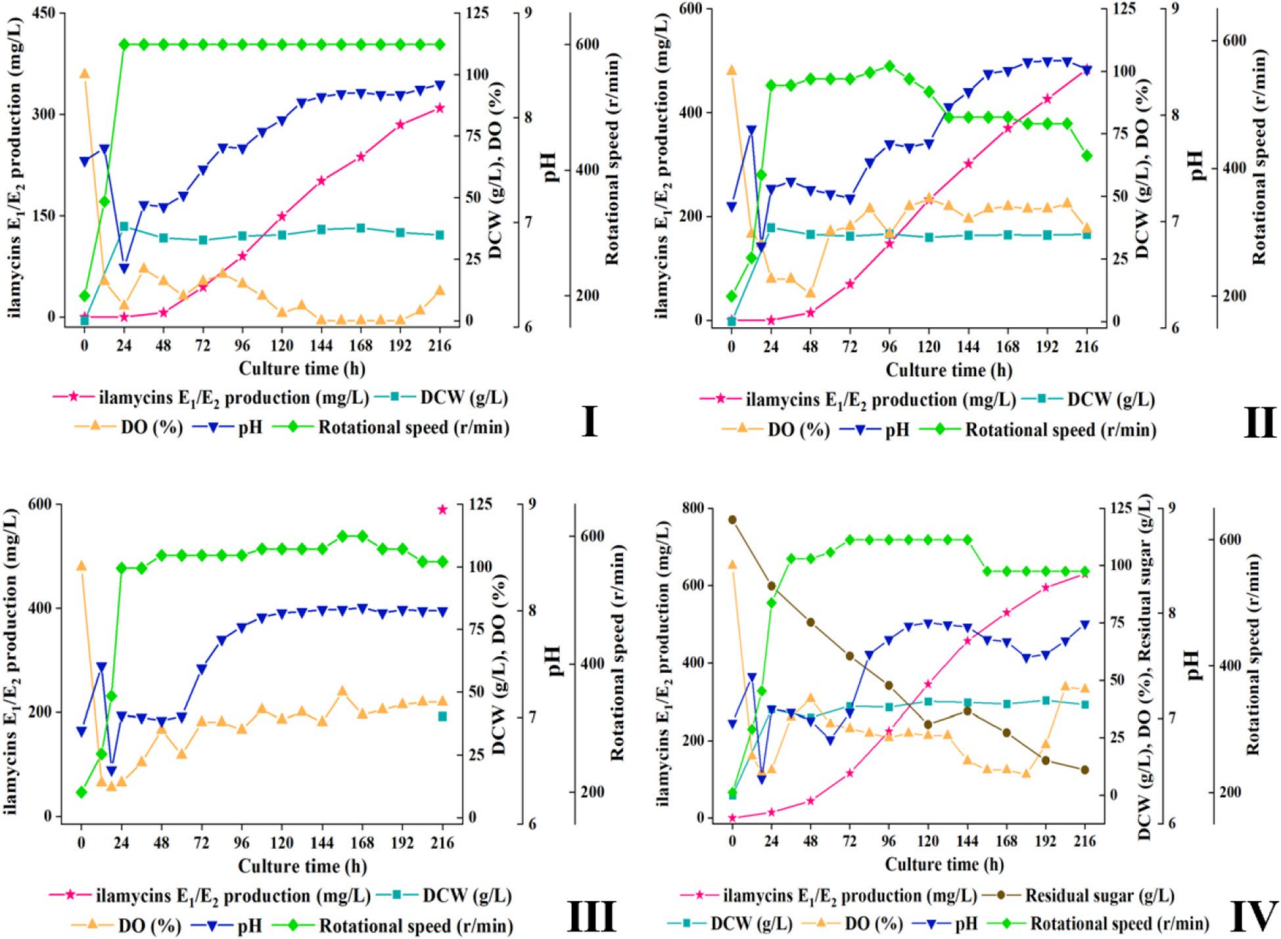
Time courses of ilamycins E_1_/E_2_ production, pH value, DCW, dissolved oxygen (DO), rotational speed and residual sugar in 5-L bioreactor (batch I–IV)

**Fig. 10:**
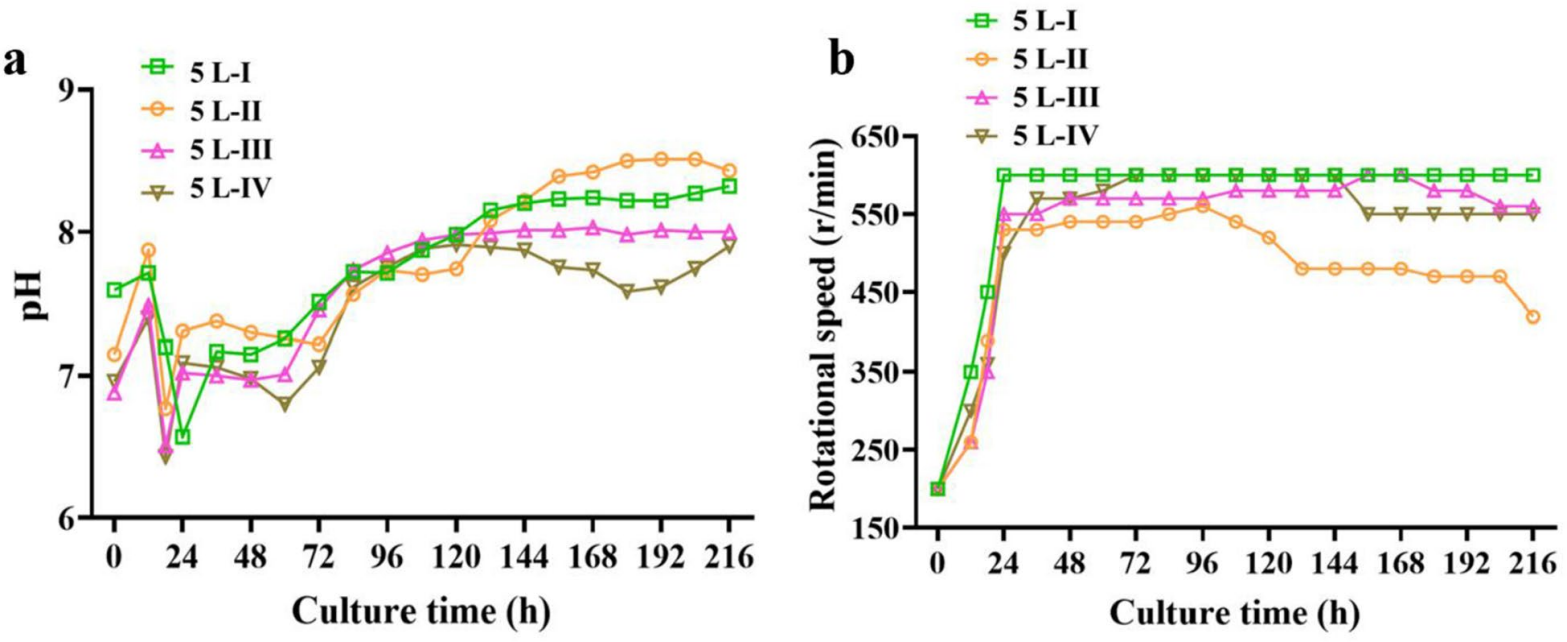
Time courses of pH values (**a**) and rotational speed (**b**) in 5-L bioreactor (batch I–IV)

**Fig. 11:**
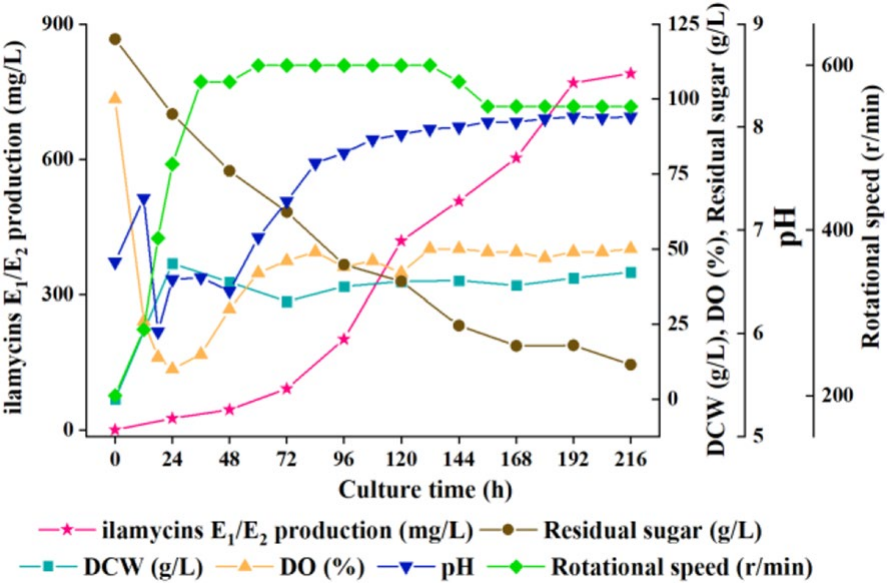
Time courses of ilamycins E_1_/E_2_ production, pH values, DCW, dissolved oxygen (DO), rotational speed and residual sugar with addition of ZnCl_2_ in a 5-L bioreactor

As shown in Figs. 9 and 10, the optimal initial pH value keeps at 6.9, and the rotational speed in the middle and late fermentation stages should also be greater than 550 rpm in 5-L bioreactor. Under these conditions, the highest production of ilamycins E_1_/E_2_ could reach 630.41 mg/L, and the total production of ilamycins was 1.15 g/L. In addition, the production of the 5-L bioreactor without sampling in batch III was not much different from that in batch IV, which proved that sampling would not cause obvious changes in the production of ilamycins E_1_/E_2_. The highest production has been obtained by adding ZnCl_2_ to the shake flask, an attempt was made to add ZnCl_2_ in the 5-L bioreactor. Adding 1 mM ZnCl_2_ solution at 0 h, the production of ilamycins E_1_/E_2_ increased to 790.34 mg/L and the total production of ilamycins was 1.48 g/L (Fig. 11).

## Conclusions

High-production medium was developed for production of novel anti-TB antibiotic ilamycins E_1_/E_2_. Exogenous addition of ZnCl_2_, tyrosine, shikimic acid, and sorbitol increased the further production of ilamycins E_1_/E_2_. A weak acid condition and mild dissolved oxygen tension is better for production of ilamycins E_1_/E_2_. Finally, the optimized fermentation process parameters were validated in a bioreactor, resulting in the highest production (790.34 mg/L) of the ilamycins E_1_/E_2_. Combinatorial strategies have potential for industrial pilot production and provide a basis for bioprocess development of novel anti-tuberculosis lead drug ilamycins E1/E2 in specific *Streptomyces* species.

### Supplementary Information

**Additional file 1: Table S1.** Seven kinds of fermentation medium. **Table S2.** Factors and levels used in Plackett–Burman design. **Table S3.** Design and results of Plackett–Burman experiments. **Table S4.** Factors and levels used in Central Composite Design. **Table S5.** Design and results of Central Composite Design. **Table S6.** Sequences of primer pairs for qRT-PCR.

## Data Availability

All data supporting this article’s conclusion are available.
