# Supplementary material for: Combinatorial strategies for production improvement of anti-tuberculosis antibiotics ilamycins E1/E2 from deep sea-derived Streptomyces atratus SCSIO ZH16 ΔilaR
Source: Bioresour Bioprocess. 2022 Oct 22;9(1):111. doi: 10.1186/s40643-022-00599-z (PMC10992044; doi:10.1186/s40643-022-00599-z)
Supplement: Supplementary file 1 — Additional file 1: Table S1. Seven kinds of fermentation medium. Table S2. Factors and levels used in Plackett–Burman design. Table S3. Design and results of Plackett–Burman experiments. Table S4. Factors and levels used in Central Composite Design. Table S5. Design and results of Central Composite Design. Table S6. Sequences of primer pairs for qRT-PCR. [file 40643_2022_599_MOESM1_ESM.docx]

**Table S1.** Seven kinds of fermentation medium.

| **Number** | **Medium Composition** |
| --- | --- |
| M1 | soluble starch 20 g/L, KNO_3_ 1 g/L, K_2_HPO_4_ 0.5 g/L, MgSO_4_·7H_2_O 0.5 g/L, NaCl 0.5 g/L, FeSO_4_·7H_2_O 0.01 g/L, yeast extract 5 g/L, sea salt 30 g/L. |
| M2 | glucose 20 g/L, peptone 2 g/L, yeast extract 2 g/L, soybean meal 5 g/L, MgSO_4_·7H_2_O 0.5 g/L, KH_2_PO_4_ 0.5 g/L, NaCl 4 g/L, CaCO_3_ 2 g/L, sea salt 30 g/L. |
| M3 | glucose 120 g/L, soybean meal 20 g/L, corn steep liquor 2.4 g/L, NaCl 6 g/L, NaNO_3_ 9.6 g/L, KH_2_PO_4_ 0.24 g/L, (NH_4_)_2_SO_4_ 6.4 g/L, CaCO_3_ 9.6 g/L. |
| M4 | glucose 20 g/L, soluble starch 10 g/L, yeast extract 10 g/L, corn steep liquor 4 g/L, MgSO_4_·7H_2_O 0.5 g/L, FeSO_4_·7H_2_O 0.1 g/L, beef extract 3 g/L, KH_2_PO_4_ 0.5 g/L, CaCO_3_ 2 g/L, sea salt 30 g/L. |
| M5 | glucose 3 g/L, corn starch 7 g/L, soybean meal 10 g/L, peptone 15 g/L, corn steep liquor 15 g/L, NaCl 8 g/L, KH_2_PO_4_ 1 g/L, NH_4_NO_3_ 6 g/L, MgSO_4_·7H_2_O 1 g/L, CaCO_3_ 5 g/L, soybean oil 4 g/L. |
| M6 | glucose 20 g/L, soluble starch 5 g/L, peptone 2 g/L, soybean meal 5 g/L, yeast extract 2 g/L, NaCl 4 g/L, K_2_HPO_4_ 0.5 g/L, MgSO_4_·7H_2_O 0.5 g/L, CaCO_3_ 2 g/L, sea salt 30 g/L. |
| M7 | soluble starch 20 g/L, peptone 2 g/L, soybean meal 5 g/L, yeast extract 5 g/L, NaCl 4 g/L, CaCO_3_ 2 g/L, sea salt 33 g/L. |

**Table S2.** Factors and levels used in Plackett-Burman design.

| **level** | **soluble starch (X_1_)** | **soybean meal (X_2_)** | **corn steep liquor (X_3_)** | **NaNO_3_ (X_4_)** | **(NH_4_)_2_SO_4_ (X_5_)** | **NaCl (X_6_)** | **KH_2_PO_4_ (X_7_)** | **CaCO_3_ (X_8_)** |
| --- | --- | --- | --- | --- | --- | --- | --- | --- |
| −1 | 96 g/L | 16 g/L | 1.92 g/L | 7.68 g/L | 5.12 g/L | 4.8 g/L | 0.192 g/L | 7.68 g/L |
| +1 | 144 g/L | 24 g/L | 2.88 g/L | 11.52 g/L | 7.68 g/L | 7.2 g/L | 0.288 g/L | 11.52 g/L |

**Table S3.** Design and results of Plackett-Burman experiments.

| **No.** | **Variable** | | | | | | | | **ilamycin E_1_/E_2_ production （mg/L）** |
| --- | --- | --- | --- | --- | --- | --- | --- | --- | --- |
|  | **X_1_** | **X_2_** | **X_3_** | **X_4_** | **X_5_** | **X_6_** | **X_7_** | **X_8_** |  |
| 1 | 1 | 1 | -1 | 1 | 1 | 1 | -1 | -1 | 49.58±15.93 |
| 2 | -1 | 1 | 1 | -1 | 1 | 1 | 1 | -1 | 76.31±16.27 |
| 3 | 1 | -1 | 1 | 1 | -1 | 1 | 1 | 1 | 199.67±23.11 |
| 4 | -1 | 1 | -1 | 1 | 1 | -1 | 1 | 1 | 101.75±12.51 |
| 5 | -1 | -1 | 1 | -1 | 1 | 1 | -1 | 1 | 192.45±9.94 |
| 6 | -1 | -1 | -1 | 1 | -1 | 1 | 1 | -1 | 176.20±20.98 |
| 7 | 1 | -1 | -1 | -1 | 1 | -1 | 1 | 1 | 154.70±25.86 |
| 8 | 1 | 1 | -1 | -1 | -1 | 1 | -1 | 1 | 129.22±13.15 |
| 9 | 1 | 1 | 1 | -1 | -1 | -1 | 1 | -1 | 96.73±31.11 |
| 10 | -1 | 1 | 1 | 1 | -1 | -1 | -1 | 1 | 182.49±28.84 |
| 11 | 1 | -1 | 1 | 1 | 1 | -1 | -1 | -1 | 122.77±31.63 |
| 12 | -1 | -1 | -1 | -1 | -1 | -1 | -1 | -1 | 152.33±27.96 |

**Table S4.** Factors and levels used in Central Composite Design.

| **Main factor** | **-1.68** | **-1** | **0** |  | **1** | **+1.68** |
| --- | --- | --- | --- | --- | --- | --- |
| A (soybean meal) | 13.28 g/L | 16 g/L | 20 g/L |  | 24 g/L | 26.72 g/L |
| B (CaCO_3_) | 1.53 g/L | 4.8 g/L | 9.6 g/L |  | 14.4 g/L | 17.67 g/L |
| C ((NH_4_)_2_SO_4_) | 0.51 g/L | 1.6 g/L | 3.2 g/L |  | 4.8 g/L | 5.89 g/L |

**Table S5.** Design and results of Central Composite Design.

| **No.** | **Variable** | | | **ilamycin E_1_/E_2_ production**  **（mg/L）** |
| --- | --- | --- | --- | --- |
|  | **A** | **B** | **C** |  |
| 1 | -1 | -1 | -1 | 94.41±18.52 |
| 2 | 1 | -1 | -1 | 112.44±8.54 |
| 3 | -1 | 1 | -1 | 135.81±12.56 |
| 4 | 1 | 1 | -1 | 178.51±24.50 |
| 5 | -1 | -1 | 1 | 117.01±8.14 |
| 6 | 1 | -1 | 1 | 147.28±11.46 |
| 7 | -1 | 1 | 1 | 193.87±17.14 |
| 8 | 1 | 1 | 1 | 232.71±17.72 |
| 9 | -1.68 | 0 | 0 | 155.40±8.49 |
| 10 | 1.68 | 0 | 0 | 184.20±15.41 |
| 11 | 0 | -1.68 | 0 | 88.31±3.25 |
| 12 | 0 | 1.68 | 0 | 247.60±2.48 |
| 13 | 0 | 0 | -1.68 | 122.31±21.22 |
| 14 | 0 | 0 | 1.68 | 185.36±17.78 |
| 15 | 0 | 0 | 0 | 195.62±14.56 |
| 16 | 0 | 0 | 0 | 180.20±8.19 |
| 17 | 0 | 0 | 0 | 209.13±11.82 |
| 18 | 0 | 0 | 0 | 197.54±7.30 |
| 19 | 0 | 0 | 0 | 200.77±13.51 |
| 20 | 0 | 0 | 0 | 203.08±8.85 |

**Table S6.** Sequences of primer pairs for qRT-PCR**.**

| **Target gene** | **Primer** | **Primer sequence (5′-3′)** |
| --- | --- | --- |
| *G6PD* | Rorword  Reverse | CGAGGTGTTCGACCCTGAC  CTCGTACATCTGGTTGGCGA |
| *6-PFK* | Forword  Reverse | GAGTCGTGCGATCCATCCTT  GGGTGACCTCTCGTTTCAGG |
| *PK* | Forword  Reverse | GTGACTGAAGTTGAAGCGGG  GCCGTTCCAAAATCGTCTGC |
| *6-PGDH* | Forword  Reverse | CTCGACAAGCTCCGTGGTT  CTTCATCTGCGGGGAGTCGT |
| *DAHPS* | Forword  Reverse | GGTCGAGGCGTTCGATGTAG  GGTCACATGGTCTGGATCGG |
| *3-DHQS* | Forword  Reverse | GAGATAGACGACGGGGTGGT  GACGACGGTGAGGAACACTT |
| *SKD* | Forword  Reverse | CATCTCCTGGCCCCGTTC  GGTGTGGAGAAGACCGACTC |
| *CS* | Forword  Reverse | GTCGATCTCGGCGACCATT  ATCGAGATCGTCAGCCACG |
| *SCO1544* | Forword  Reverse | GTACATCGAGGCGGTTCTCG  ATGTGGCGTCCGTCGATCA |
